# Physiologic and Clinical Principles behind Noninvasive Resuscitation Techniques and Cardiac Output Monitoring

**DOI:** 10.1155/2012/531908

**Published:** 2011-08-16

**Authors:** Anthony M. Napoli

**Affiliations:** Department of Emergency Medicine, Brown University Medical School, 593 Eddy Street, Davol 142, Providence, RI 02903, USA

## Abstract

Clinical assessment and vital signs are poor predictors of the overall hemodynamic state. Optimal measurement of the response to fluid resuscitation and hemodynamics has previously required invasive measurement with radial and pulmonary artery catheterization. Newer noninvasive resuscitation technology offers the hope of more accurately and safely monitoring a broader range of critically ill patients while using fewer resources. Fluid responsiveness, the cardiac response to volume loading, represents a dynamic method of improving upon the assessment of preload when compared to static measures like central venous pressure. Multiple new hemodynamic monitors now exist that can noninvasively report cardiac output and oxygen delivery in a continuous manner. Proper assessment of the potential future role of these techniques in resuscitation requires understanding the underlying physiologic and clinical principles, reviewing the most recent literature examining their clinical validity, and evaluating their respective advantages and limitations.

## 1. Background

Consensus recommendations, research, and meta-analyses have all questioned the efficacy of routine use of pulmonary artery catheterization in critically ill patients [1–3]. Other studies have questioned its accuracy, with limits of error of ±20–22% [4, 5]. Early adaptation of invasive protocols utilizing arterial and central venous access has become the standard in some cases [6] despite the limitations of some components of such protocols [7]. Research has long since demonstrated that traditional parameters of hemodynamic stability (heart rate and blood pressure) are poor predictors of the degree of cardiac dysfunction [8] or organ failure [9] and that physicians are poor predictors of hemodynamics in critically ill patients [10–12]. As much as 18% of “hemodynamically stable” sepsis patients (blood pressure > 90 mmHg, lactate < 4 mmol/L) can progress to severe sepsis and septic shock within 72 hours [13]. 

Noninvasive techniques for measuring hemodynamic variables have existed for some time; older techniques are being refined and newer techniques are being developed. Most research to date has focused on the validation of these technologies with little demonstrated efficacy, and there is a lack of multicenter trials pairing these technologies to dedicated treatment protocols [14]. Noninvasive hemodynamic monitoring and guided resuscitation has the potential to offer critical care clinicians information that is clinically compatible with pulmonary artery catheterization and potentially provide such information earlier and in a safer manner than traditional parameters used in resuscitation. The objective of this paper is to discuss the principles behind the clinical development of newer completely noninvasive techniques to assess preload dependence and the hemodynamic monitors that measure cardiac output and oxygen delivery that are available for care of the critically ill patient.

## 2. Preload Dependence

The Frank-Starling relationship is curvilinear; a steep and nearly linear relationship exists in the volume dependent ascending portion of the Frank-Starling curve where stroke volume (SV) is intimately related to preload, termed preload dependence [15–17]. Preload independence occurs as the Frank-Starling curve plateaus. Fluid responsiveness (FR) is generally defined as an increase of 10–15% in stroke volume (SV), cardiac output (CO), or cardiac index (CI) in response to volume expansion and indicates unmasked hypovolemia or preload dependency [18]. 40–72% of critically ill patients respond to volume expansion with a change in stroke volume, suggesting a need for better methods of predicting fluid responsiveness [19]. The discussion that follows details the limitations of static measures of fluid responsiveness and the advantages of dynamic measurement of preload dependency.

### 2.1. Static Measures of Fluid Responsiveness

#### 2.1.1. Central Venous Pressure

Central venous pressure (CVP), the most common static measure of preload, is clinically estimated by transducing the pressure in a central venous line. It serves as an important component of the management of critically ill patients [6]. However, static measures such as central venous pressure and pulmonary capillary wedge pressure are poor measures of cardiac preload and do not predict response to fluid therapy [20]. A meta-analysis of studies examining the ability of CVP to predict fluid responsiveness reported a poor association (*r*  =  0.18, ROC  =  0.56) and concluded “CVP should not be used to make clinical decisions regarding fluid management” [7]. CVP as a tool for predicting FR is limited because it does not account for whether the patient is in a preload dependent or preload independent part of the Frank-Starling curve. Another major limitation is that one cannot adequately account for the degree to which transmitted pressures from comorbidities (i.e., cor pulmonale) or clinical conditions (i.e., mechanical ventilation) may affect the accuracy of measurement. The ability of CVP to predict fluid responsiveness is also altered by differences in ventricular compliance or changes in ventricular contractility. A young individual with a compliant ventricle may be volume overloaded at the same central venous pressure of an older individual with a stiff ventricle who may be volume depleted. Ventricular contractility can be impaired in conditions like sepsis where a rightward and downward shift in left ventricular stroke work is present at a given volume [21–23]. The flatter curve that results from depressed LV systolic function limits the exploitation of the Frank-Starling relationship of improving stroke volume by improving preload. 

CVP does, however, represent an opportunity to estimate the right atrial pressure (RAP) and the pulmonary artery pressure (PAP). CVP, measured by sonographic diameter of the inferior vena cava (IVC), is nearly equivalent to the RAP and is a fair estimate of the PAP if the peak tricuspid regurgitant flow (*V*_peakTR_) is minimal:

(1) $$\text{CVP}\approx \text{RAP},\;\;\text{PAP}=\text{RAP}+4*{\left(V_{\text{peakTR}}\right)}^{2}.$$

This technique accurately reflects pulmonary artery pressure and performs much better than estimates of jugular venous distension [24]. The RAP can be estimated (Table 1) by utilizing the known relationship between RAP and inferior vena cava (IVC) diameter and the caval index (the fractional change in the IVC diameter during respiration) [25, 26]. A greater than 50% decrease in IVC diameter is associated with a CVP <8 mmHg (*r*  =  0.74) in the early stages of resuscitation from severe sepsis [27]. The main limitation of this technique in spontaneously breathing patients is that certain clinical situations inducing changes in intrathoracic pressure (i.e., asthma, emphysema) may cause changes in IVC diameter that are more reflective of these intrathoracic pressure changes than they are preload responsiveness [28, 29]. Alternatively, the absence of respiratory variations in IVC diameter in these conditions is generally indicative of the absence of preload responsiveness. The accuracy of this technique is also dependent upon the IVC sampling location and the interrater reliability of this approach has yet to be established [30]. Thus, this represents an early adjunct in the management of acutely ill patients that is both feasible and safe but needs further validation. Recent small studies have suggested that respiratory changes in inferior vena cava diameter may be helpful as a dynamic measure of predicting FR in mechanically ventilated sepsis patients [31–33]. 

Other static measures of assessing preload have been studied. However, these methods generally require invasive monitoring (like right atrial pressure, pulmonary artery pressure, or the intrathoracic blood volume index) or techniques, like right ventricular end diastolic volume by echocardiography, that require skills not maintained by most critical care clinicians [18, 34].

### 2.2. Dynamic Measures of Fluid Responsiveness

Dynamic parameters can predict an increase in cardiac output from volume expansion before such volume expansion is performed and are better predictors of FR than static parameters [19]. Understanding how dynamic measures are predictors of fluid responsiveness requires an understanding of how respiratory variation can impact the preload, pulse pressure, and stroke volume. Spontaneous inspiration leads to a larger venous return to the right side of the heart but also leads to the displacement of the septum and pulmonary vein dilatation leading to reduced preload to the left side of the heart. This reduced preload results in less ventricular filling and a lower left ventricular stroke volume. Expiration leads to decreased intrathoracic pressure, higher preload, and larger stroke volumes on the left side of the heart. This increase in left cardiac preload occurs at expiration as a result of the transmission of the increase in right cardiac preload after the lung transit time. Such changes in pulse pressure during spontaneous respiration are otherwise known as pulsus paradoxus. Changes in intrathoracic pressure from mechanical ventilation can lead to cyclic changes, but reversed—otherwise termed reverse pulsus paradoxus. These small changes in right ventricular preload induced by mechanical ventilation can lead to significant changes in stroke volume in the ascending part of the Frank-Starling curve (i.e., in preload-dependent individuals). On the left side of the heart, if arterial compliance is constant through a respiratory cycle then variations in systolic blood pressure and pulse pressure (PP) will be reflected in left ventricular stroke volume [35]:

(2) $$C=\frac{dV}{dP}=\frac{\Delta V}{\Delta P}=\frac{\text{SV}}{\text{PP}},\;\;\text{SV}=C\times \text{PP}.$$

#### 2.2.1. Pulse Pressure Variation

Clinical study has long since established the relationship between systolic pressure variation (SPV) and FR [36, 37]. While SPV and pulse pressure variations (PPV) in mechanically ventilated patients are predictive of FR [38, 39] in sepsis patients, PPV appears to be the better measure [39]. PPV is typically represented as a percent:

(3) $$\text{PPV}=100*\frac{\left({\text{PP}}_{\max\;}-{\text{PP}}_{\min\;}\right)}{\left(\left({\text{PP}}_{\max\;}+{\text{PP}}_{\min\;}\right)/2\right)}.$$

PPV is highly predictive of FR with cutoff values of 11–13% according to various studies [40, 41]. Traditional measurement of pulse pressure on a beat-to-beat basis has required arterial cannulation. However, recent literature comparing the *noninvasive *pulse oximetry waveform amplitudes to standard arterial cannulation has shown that respiratory variations in the noninvasive pulse oximetry waveform (ΔrPOP) have a high correlation with PPV (*r*  =  0.83) in mechanically ventilated patients [41]. A ΔrPOP >15% roughly equates to PPV >13%. These results were validated in operating room [42] and postoperative cardiac surgery patients [43]. Clinical study has been promising [41, 44]; ΔrPOP with a cutoff of 13% predicted FR with a sensitivity of 93% and specificity of 90% [41]. Technology equipped with the capacity to calculate ΔrPOP would substantially improve the resuscitation of mechanically ventilated critically ill patients by providing a simple and noninvasive method of predicting FR. However, while the potential of a noninvasive measure like ΔrPOP supplanting arterial PPV measurement is promising, its use is limited to mechanically ventilated patients.

#### 2.2.2. Stroke Volume Variation

Stroke volume variation (SVV) is believed to be less affected by vasomotor tone then PPV and is, therefore, likely to be a better measure of FR in mechanically ventilated patients [45, 46]. A study comparing SVV to pulmonary artery catheterization by thermodilution (PAC-TD) demonstrated SVV (ROC  =  0.82) to be equivalent if not better than PPV (ROC  =  0.80) in mechanically ventilated patients [47]. In both studies, CVP performed poorly as a measure of preload responsiveness [46, 47]. Most noninvasive hemodynamic monitors (discussed below) can measure stroke volume variation by way of the arterial pressure curve. SVV, much like PPV and ΔrPOP, is limited to mechanically ventilated patients, as preload is highly susceptible to changes in intrathoracic pressure. 

Despite the promising results of studies of PPV and SVV in mechanically ventilated patients, limitations exist in these methodologies. In mechanically ventilated patients, a linear relationship exists between tidal volume and SVV or PPV [48]; tidal volumes of less than 8 mL/kg are no more accurate than traditional measures of preload [49]. Recent work has demonstrated that lower tidal volumes, impaired contractility, or elevated respiratory rates each independently result in lower SVV and PPV errantly leading to an increase in falsely negative tests for fluid responsiveness [50, 51]. Meanwhile, right ventricular dysfunction may cause a false positives PPV [52], a result that could lead to volume overload and deleterious affects in certain populations [53]. Interestingly, increased contractility does not influence PPV or SVV [50]. Early inspiratory augmentation of left ventricular stroke volume (often termed dUP) and irregular cardiac rhythms may affect the reliability of these parameters as well [38, 45].

#### 2.2.3. The Preejection Period

The preejection period (PEP), the time between onset of ventricular depolarization and ventricular ejection, is a systolic time interval believed to be representative of contractility. As the Frank-Starling curve would indicate, the lower the ventricular preload the shorter the PEP. Respiratory changes in the preejection period (*r*PEP) are an accurate measure of FR in septic ventilated patients [54]. PEP, like SVV, can be measured by some currently available noninvasive monitors. However, *r*PEP has not been studied using noninvasive monitoring partly because only some noninvasive monitors simultaneously record the electrocardiogram (ECG) and the arterial pressure waveform. A noninvasive method of measuring FR in both spontaneously breathing patients and mechanically ventilated patients is needed.

#### 2.2.4. Passive Leg Raising

Passive leg raising (PLR) is a simple, reversible maneuver that mimics a rapid volume expansion of approximately 300–500 mL [55, 56] by shifting blood from the lower extremities to the core venous circulation [57, 58]. A standard PLR is done by placing a patient in a semirecumbent position for three minutes then laying the patient supine with the legs elevated to 45 degrees for three minutes. Fluid responsiveness is generally defined as a change in cardiac index (ΔCI), cardiac output (ΔCO), or stroke volume (ΔSV) of >10–15% [18]. PLR-induced changes in SV represent a good predictor of fluid responsiveness independent of breathing conditions [56, 59–61]. A recent meta-analysis demonstrated PLR-induced changes in cardiac output was more accurate than measured changes in arterial pulse pressure, with a pooled sensitivity, specificity, correlation, and area under the receiver operator curve of 89%, 91%, 0.81, and 0.95, respectively [62]. These studies validate for the first time a technique for noninvasively measuring fluid responsiveness in ventilator-dependent and spontaneously breathing patients.

#### 2.2.5. Utilizing Dynamic Measures of Fluid Responsiveness

Passive leg raising matched with a noninvasive technology to assess hemodynamic response would be an important advance in critical care settings where traditional management methods of critically ill individuals may not be immediately available in early resuscitation (such as measuring CVP and ScVO_2_), of suspect accuracy (CVP in determining preload), or functionally inaccessible (i.e., hemodynamics measured by thermodilution). 

Minimizing respiratory variations in stroke volume by volume loading is a good surrogate for maximizing cardiac output by volume expansion until patients reach the preload independent part of the Frank-Starling curve. However, measuring fluid responsiveness still has significant limitations. The most obvious limitation is that all of these dynamic measures of FR, with the exception of PLR, are limited to mechanically ventilated patients, ideally with tidal volumes of at least 8 mL/kg [49]. Other limitations include unstable or irregular rhythms, the necessity of a sealed chest cavity, and a normal abdominal compartment pressure. ΔrPOP is being integrated into the monitoring systems of currently available standard monitors, and it comes at a limited cost; however, its efficacy has yet to be demonstrated outside the operating room [63]. Like CVP, it is a continuous and easily interpretable number. However, none of the measures of FR provide the critical care clinician with other hemodynamics (such as cardiac output or oxygen-carrying capacity) which may be particularly helpful in managing undifferentiated shock, shock of more than one etiology, and nonfluid responders. PLR and SVV are measureable on noninvasive hemodynamic monitors, though the latter is limited to mechanically ventilated patients. Maximal effect of PLR occurs within the first minute, making it important to assess this change in conjunction with real-time stroke volume and cardiac output monitors.

## 3. Noninvasive Hemodynamic Monitoring

Multiple methodologies for noninvasive hemodynamic and resuscitation monitoring are available in the management of acutely ill patients in the ED. Each technology offers a unique set of advantages and limitations (summarized in Table 2). Literature questioning the efficacy of routines use of PAC-TD, it's limitations of accuracy, and the desire for noninvasive alternatives have led to wider consideration of these monitors. Bedside ultrasonography, transcutaneous doppler ultrasonography, thoracic bioimpedance, and bioreactance represent some of these alternatives discussed below. 

### 3.1. Bedside Ultrasonography

**Figure 1:**
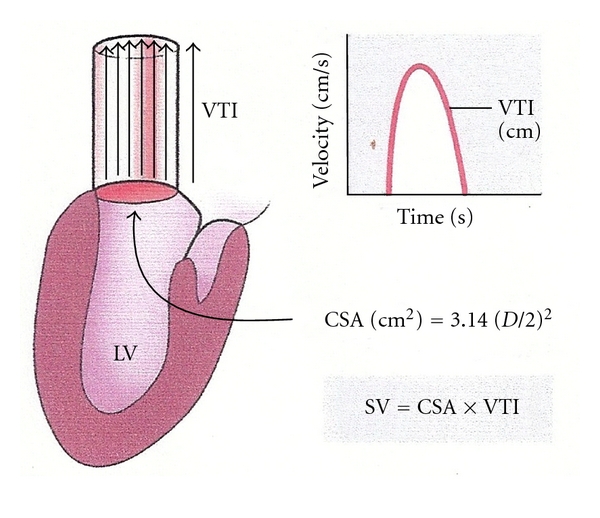
Doppler assessment of stroke volume via the left ventricular outflow tract. This illustration demonstrates the physiologic principle of measuring stroke volume by measuring the LVOT diameter and the VTI at that point. A characteristic curve is generated by proper placement of a probe in line with the flow of blood out of the LVOT. This figure was published in the Textbook of Clinical Echocardiography, 3rd edition, Elsevier, Ltd, 2004. Page 147, Used with permission [25].

The use of bedside ultrasonography in critically ill patients is becoming more frequent as technical advances, cost reductions, and safety concerns have led to many EDs and intensive care units (ICU) having bedside portable ultrasound. While volumetric methods of measuring stroke volume exist (termed the method of discs or Simpson's rule), a more reliable measure of cardiac output comes from simple physics. Flow is the product of the velocity (*V*) of a fluid moving through a certain location and the cross sectional area (CSA) of that location. Therefore, the stroke volume can be measured by calculating the velocity of blood emerging from the left ventricular outflow tract (Figure 1), such that

(4) CO=HR∗SV,

(5) $$\text{SV}=\text{VTI}*\text{CS}{\text{A}}_{\text{LVOT}}.$$

This technique is a validated, accurate measure of stroke volume that is used extensively in echocardiography [64, 65]. Physicians attempting to utilize this technique must be facile in identifying the left ventricular outflow tract, measuring the time integral of velocity through the region (VTI), and obtaining a 5-chamber apical view—all of which are difficult to do in critically ill patients. One generally measures VTI by placing a Doppler probe at the suprasternal notch and aiming it directly opposite the direction of blood flow. This technique has been utilized extensively in echocardiography and is the basis for transcutaneous doppler ultrasonography (TCDU).

Despite the difficulty in using ultrasound for the measurement of stroke volume and cardiac output, focused critical care echocardiography is increasingly being recognized as an important adjunct in the care of critically ill patients because of the wealth of information one can obtain. Multiple studies have now shown that short training programs can adequately train novice users in the necessary skills for bedside ultrasonography [66–69]. These skills include assessment of global left ventricular function, ventricular size, inferior vena cava diameters, and identifying pericardial effusions and tamponade [67, 69–72]. Assessment of right ventricular function by echocardiography is important in certain types of shock and may contribute to limited responses to fluid resuscitation [73, 74]. While transabdominal inferior vena cava collapsibility index is a promising measure of fluid responsiveness and central venous pressure [27, 32, 33], transesophageal echocardiography also offers the potential to measure superior vena cava collapsibility and may be one of the best measures of fluid responsiveness [75, 76].

#### 3.1.1. Advantages

Skilled sonographers can utilize enhanced echocardiography to examine wall motion abnormalities and valvular disease. With proper training, one can measure SV and CVP, make qualitative assessments of ventricular function, as well as evaluate primary and secondary etiologies of hemodynamic instability (i.e., pericardial tamponade). For these reasons, independent of hemodynamic measurement, bedside ultrasonography has an important role in the resuscitation of critically ill patients.

#### 3.1.2. Limitations

The greatest limitation of bedside ultrasonography is that it is discontinuous. Findings may lead to definitive changes in management but to utilize this technology of as a hemodynamic monitor or to monitor therapeutic effectiveness requires regular reevaluation. While multiple studies have now demonstrated the validity of short training courses and curricula for novice users, few studies currently exist that demonstrate the therapeutic impact of this skill set [71]. 

### 3.2. Transcutaneous Doppler Ultrasonography

**Figure 2:**
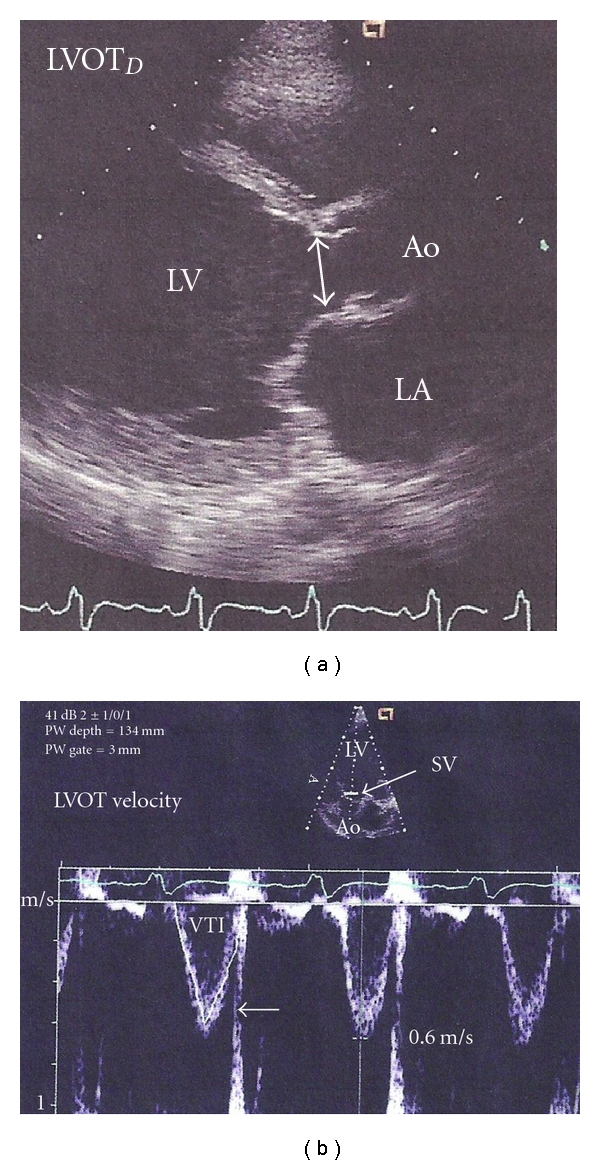
Transducer placement and characteristic waveform using transcutaneous doppler ultrasonography. This demonstrates the characteristic transducer direction and image capture using the direct measurement through the left ventricular outflow tract. One notes a characterstic waveform and manipulates the transducer to obtain maximum outflow. This figure was published in the Textbook of Clinical Echocardiography, 3rd edition, Elsevier, Ltd, 2004. Page 148, Used with permission [25].

Transcutaneous wave doppler ultrasonography (TCDU) is an extension of bedside echocardiography that is based on the same principle of measuring the VTI at the left ventricular outflow tract (LVOT). The relationship in cadaver studies between height and a normal LVOT diameter obviates the need to manually measure the LVOT diameter. The provider obtains the stroke volume by obtaining the patient's height and measuring the VTI using visual and auditory cues to improve positioning of a transcutaneous Doppler probe (Figure 2).

There appears to be a high correlation (*r*  =  0.87) with PAC-TD, with minimal bias and limits of agreement of approximately ±1 L/min [77, 78]. Studies have demonstrated excellent interrater reliability of this technology with minimal to no training [79–81]. The corrected aortic flow time (FTc), a Doppler measure of the duration of flow during systole corrected for heart rate, has been used in conjunction with TCDU and appears to be an effective measure of FR in spontaneously breathing patients in septic shock [82]. TCDU can also predict FR when used in conjunction with PLR in critically ill patients [83].

#### 3.2.1. Advantages

Transcutaneous doppler ultrasonography is fast, easy to learn, and has high interrater reliability even in novice users. While the initial cost of this technology is comparable with others, one of its major advantages is that there are no per-patient costs associated with disposable parts. Measurements are obtained in the suprasternal notch so there is minimal interference with patient care and patient specific factors like obesity, diaphoresis, or positioning have limited effect on the accuracy of the results. 

#### 3.2.2. Limitations

The largest limitation of this technology is that continuous monitoring is not possible. Though there are no studies demonstrating that continuous hemodynamic monitoring improves outcome in critically ill patients, non-continuity of hemodynamic monitoring requires clinicians to decide on a preset protocol of recurrent measurement or to utilize this technology when clinical circumstances dictate. Since physicians are poor predictors of underlying hemodynamic instability, a noncontinuous monitoring technique may help direct treatment when combined with a protocol but it likely will not help alert clinicians to hemodynamic decline. Additionally, because the LVOT diameter is assumed, anatomical changes such as aortic valve regurgitation, aortic valve stenosis, or proximal aortic aneurysms/dilatation can cause significant alterations in the accuracy of the stroke volume. Though clinical exam can detect many of these abnormalities, it is not uncommon for patients to have clinically indetectable stenosis, regurgitation, or aneurysms—particularly in the critically ill. Lastly, clinical trials have largely been validations against other hemodynamic monitoring techniques or evaluations of inter-rater reliability. Few studies exist that compare this technology to other methods of measuring hemodynamics in critically ill patients; those that do question the device's accuracy [84].

### 3.3. Impedance Cardiography or Thoracic Bioimpedance

**Figure 3:**
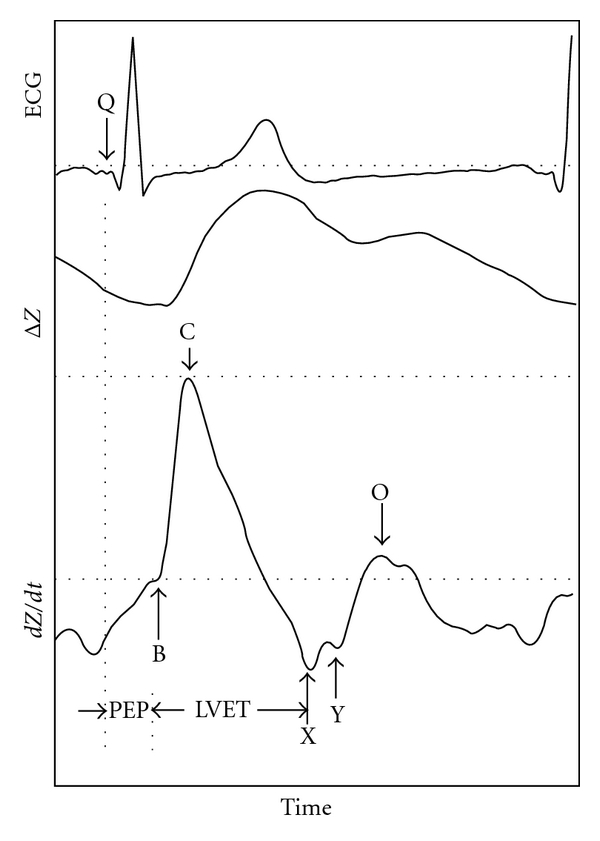
Characteristic waveforms for thoracic bioimpedance monitoring. Product photographs reprinted with permission from SonoSite; _electrical and mechanical change in impedance over change in time. Trademarks and logos are trademarks owned by SonoSite, Inc. PEP: preejection period, LVET: left ventricular ejection time,* ΔZ*: change in impedance, *dZ/dt*: 1st derivative of impedance waveform.

Impedance cardiography (ICG) or thoracic bioimpedance (TEB) is a noninvasive means for obtaining continuous hemodynamic data. This technology has been validated in over 2000 patients in multiple different settings against multiple different gold standards [85]. It is based on measuring changes in thoracic impedance of high-frequency, low-magnitude alternating currents applied across the thorax. The impedance (*Z*) of various tissues across the chest can change with time, but the blood in the aorta is the only component in the thorax that changes over a few seconds' time. Pulse waves will, therefore, naturally travel down the aorta and be responsible for the beat-to-beat alterations in impedance, providing characteristic waveforms that can be used to calculate hemodynamic variables (Figure 3). 

**Figure 4:**
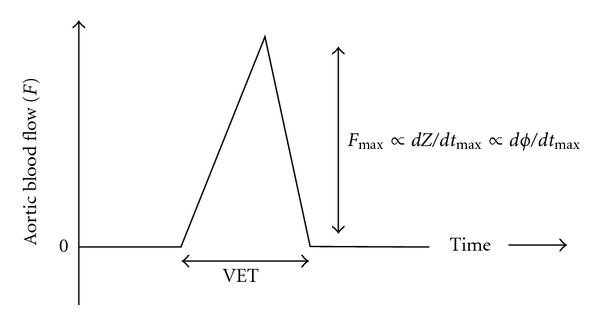
Determining stroke volume using thoracic bioimpedance or bioreactance. Schematic representation of stroke volume measured from aortic blood flow by measuring the change in impedance (*dZ*/*dt*_max_) using thoracic bioimpedance or the relative phase shift (*dϕ*/*dt*_max_) using bioreactance. These parameters are representative of the peak blood flow, and the stroke volume is proportional to the product of this parameter over the ventricular time (VET) for each device, respectively [92].

The area under the curve of the pulse pressure waveform represents the stroke volume. Typically, this area can be estimated by the peak pressure change during systole multiplied by the ventricular ejection time (VET), the time between opening and closing of the aortic valve. Using bioimpedance, one can estimate the stroke volume by measuring peak change in impedance (*dZ*/*dt*_max_) multiplied by the VET (Figure 4). Measurement of *dZ*/*dt*_max_ requires a pair of thoracic impedance leads and measurement of VET requires the acquisition of an ECG signal; two sets of leads are necessary—each with an adequate signal. A change in impedance between two sets of leads requires measuring the amplitude of the impedance signal at each lead *and* measuring the distance between the leads because the amplitude degrades over time. More recent research has enhanced the ease and accuracy of monitoring by (1) assuming the thorax is a cylinder or cone, (2) which makes up 17% of one's overall height (*H*), (3) and can be normalized by ideal body weight [86]. Therefore, the stroke volume is calculated as

(6) $$\text{SV}=c\left(\frac{{\left(0.17*H\right)}^{3}}{4.2}\right)*\frac{dZ}{dt_{\max\;}}*\frac{\text{VET}}{Z}.$$

TEB also allows for measuring central thoracic fluid volume by assessing the overall impedance (*Z*), as well as measuring systolic time intervals and the accelerated cardiac index, all measures that may have use in certain clinical situations [85, 87–89]. Early research was promising but inadequate for advocating routine use of TEB as a surrogate for PAC-TD because of the large limits of agreement [90]. Overtime algorithms have improved; a large multicenter study of over 2000 measurements in 861 critically ill ED, ICU, or operating room patients found a good correlation (*r*  =  0.85) with a bias of −0.12  +  0.75 L/min/m^2^ [91]. This study represents the single largest multicenter study to validate a noninvasive hemodynamic monitor in critically patients. Weighted average and meta-analytic correlation coefficients comparing TEB with other methodologies of measuring cardiac output demonstrates correlations ranging from *r*  =  0.61 (Doppler Echocardiography) to *r*  =  0.89 (left ventricular assist device) with an overall correlation of *r*  =  0.81 (*n*  >  16,000) and the same correlation (*r*  =  0.81) in studies comparing TEB with PAC-TD (*n*  =  10,959). The largest single study in critically ill patients found the percent limit of agreement (LOA) between TEB and PAC-TD to be 16.6% with even better performance (9.8%  ±  6.7%, *r*  =  0.93) when motion artifact and clinical conditions affecting the uniformity of thoracic impedance are accounted for [91]. 

#### 3.3.1. Advantages

TEB technology offers the clinician a well-studied method to noninvasively, continuously monitor hemodynamics. The capacity for beat-to-beat measurement of the impedance waveform allows for a more accurate and responsive measurement of stroke volume as the measurement period can be selected by the user. TEB has the largest and widest breath of literature validating its accuracy. It also remains the only technology that has been validated, at least in part, in critically ill patients.

#### 3.3.2. Limitations

Morbid obesity, a short neck, extensive hair, diaphoresis, and inability to localize landmarks are limitations. Clinical conditions such as pneumonia, pleural effusions, hemo/pneumothorax, or significant third spacing from late stage sepsis may also impair the accuracy [85]. Multiple small studies have also questioned the accuracy of TEB in measuring CO of mechanically ventilated patients during PEEP [94–96]. Improper placement or errors in the assumed relationship between height and thoracic length can lead to significant errors due to the mathematical relationship (7) used to determine stroke volume. Despite these limitations, inter-rater reliability is very good [81]. These known limitations and the continued variability in reports of the accuracy of this technology, particularly in critically ill subsets, have hindered the adoption of the technology.

### 3.4. Bioreactance

Bioreactance is the newest technique for noninvasive hemodynamic monitoring. Bioreactance (BR) is very similar to TEB in that, an electrical current of low amplitude and known frequency is applied across the chest between two leads. The difference between the two methodologies is analogous to the AM (amplitude modulated) radios (TEB) and FM (frequency modulated) radios (BR) of impedance hemodynamics. As such, the frequency of a signal does not degrade with distance, and the ability to record adequate signal intensity, therefore, becomes independent of distance. BR measures the relative phase shifts in the applied and received signal between two leads that is created by changes in intrathoracic volume resulting from blood flow. These phase shifts are also generally less susceptible to signal degradation and more amenable to high-pass filtering to eliminate ambient noise, theoretically leading to more accurate signal recovery. As opposed to TEB (7), SV with BR is not a function of the distance between leads but simply a function of (VET), the maximum phase shift over time (*dϕ*/*dt*_max_), and a constant (*c*) (8).

(7) $${\text{SV}}_{\text{TEB}}\approx {\left(\frac{L}{Z}\right)}^{2}*\text{VET}*\frac{dZ}{dt_{\max\;}},$$

(8) $${\text{SV}}_{\text{BR}}\approx c*\text{VET}*\frac{d\phi }{dt_{\max\;}}.$$

While this technology is much newer than TEB, recent literature on the accuracy of this monitoring technique against TD and other hemodynamic monitoring is as strong if not better [92, 97, 98]. The largest single-site validation of a noninvasive hemodynamic monitor, a study of 110 consecutive postcardiac surgery patients with a total of 65,888 paired measurements between BR and PAC-TD, demonstrated a correlation of *r  *=  0.82 with a bias of 0.16  ±  0.52 L/min (which equated to a percent bias of 4  ±  11.3%) [98]. A subsequent multicenter validation of BR versus PAC-TD resulted in a similar correlation (*r  *=  0.78) [97] with limits of agreement that were similar to those observed in prior studies of continuous versus bolus PAC-TD [99]. Early results in clinical studies in patients with septic shock that compare BR to PAC-TD suggests that the accuracy and precision are maintained in both baseline measurements (*r  *=  0.88) and during PLR testing (bias 6.8  ±  13%) [100].

#### 3.4.1. Advantages

The most important advantage of BR is that frequency modulations and phase shifts are independent of the distance between the applied and detected signal. Lead placement using BR requires neither an exact distance nor an exact location on the thorax. This allows for more convenience and less interference of lead placement. Additionally, anatomic or clinical conditions such as obesity, short neck, and diaphoresis do not degrade the accuracy of a frequency-modulated signal. Measurements are not affected by other voltage sources and known frequency interference (such as ambient noise) can be filtered out.

#### 3.4.2. Limitations

This technology is fairly new, but there are few currently identifiable disadvantages. The validation literature is very strong though there are few published clinical studies to date utilizing this technology in undifferentiated or specific critically ill patient populations. TEB has a much broader experience in clinical use though its role in specific conditions or undifferentiated critically ill patients remains controversial because of the reports of variable accuracy [85, 90].

## 4. Conclusion and Future Directions

### 4.1. Per-Beat Hemodynamic Measurement

Expressing patient hemodynamics as a per-minute phenomenon is not reflective of the basic function of the cardiovascular system—to respond to ongoing metabolic demands with immediate changes in oxygen delivery. As such, per-minute hemodynamic measurement may not be reflective of ongoing changes particularly in hemodynamically unstable individuals. In spontaneously breathing patients, the thoracic pressure changes in concert with the frequency of respirations leading to varying rates of changes in thoracic pressure. Even in a steady state, 20–30% of beat-to-beat variations in heart rate and stroke index have been demonstrated [93]. While standard convention is to extrapolate measurements and represent them on a per-minute basis, measurements are not typically taken over one minute. The actual time period of measurement and consequently the number of respirations during that time are likely to be different depending on the methodology; thermodilution is measured over a set period of time while many of the noninvasive technologies use a different time period or measure over a running several beat average. As such, newer noninvasive technology may be more reflective of ongoing hemodynamic response to clinical conditions.

### 4.2. Modeling the Hemodynamic Response

**Figure 5:**
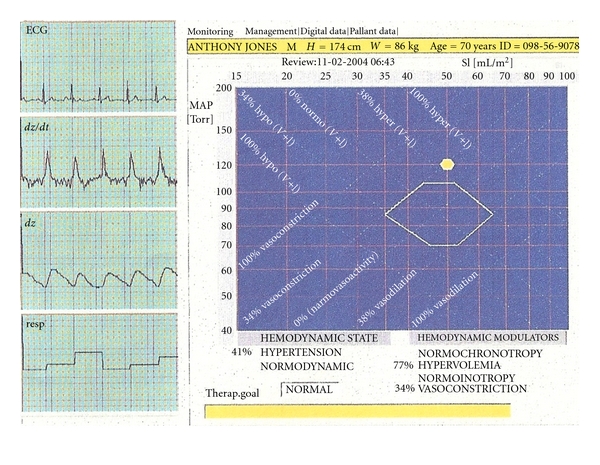
Graphical representation of the normalized hemodynamic modulators that determine the hemodynamic state. This is a graphical illustration of the four hemodynamic modulators (preload, afterload, inotropy, and chronotropy) that determine one's hemodynamic state. Measurement on a per-beat basis, and normalization of each measure allows for percentile representation of each modulator effect with respect to the whole [93].

If the hemodynamic parameters of volume, inotropy, chronotropy, and vasoactivity can be measured noninvasively and the normal values for these hemodynamic parameters are known, then these parameters can be normalized and the hemodynamic state of each patient can be graphically represented by superimposing these parameters on the same graph and representing them as percent changes from the norm (Figure 5). This would allow graphical modeling of the hemodynamic state, and clinicians may be able to more accurately respond to the individual needs of the patient with targeted therapy. Clinicians would not need to continuously interpret the per-beat hemodynamic variables within the context of “normal” or patient body habitus. Thus, measurement of these hemodynamic parameters, indexing them by body surface area, and analyzing them all in concert may be important in the future monitoring of critically ill individuals. This technique was first proposed by Sramek et al. and has demonstrated improved outcome in antihypertensive management but has yet to be studied in critically ill patient populations [93, 101–103].

### 4.3. Outcomes-Based Research

While the noninvasive technology described here and the physiologic principles underlying this technology may initially be difficult to understand, this technology may be the next step in improving the resuscitation of the critically ill. Algorithms based on noninvasive hemodynamic parameters that better characterize the body's hemodynamic response to illness may be more intuitive and can potentially be applied in a larger breath of patient population. Measures of FR would improve upon measuring CVP and stroke index could augment ScVO_2_ in management strategies like early goal directed therapy for sepsis. Most of the noninvasive hemodynamic monitoring literature to date has focused on single or multi-center validations of device accuracy, proofs of concept, or observational trials of the prognostic capacity of individual hemodynamic parameters. Future research will undoubtedly necessitate a multicenter trial utilizing hemodynamic monitoring to drive clinical interventions in protocols for the resuscitation of critically ill patients. The noninvasive hemodynamic techniques discussed here may provide the means to improve upon aggressive resuscitation while other new techniques for noninvasively measuring tissue level response to resuscitation may aid us in knowing when those efforts have succeeded [104–106].

## Figures and Tables

**Table 1:** Central venous pressure by ultrasonography of the inferior vena cava.

IVC diameter (cm)	Respiratory collapse	RA pressure (mmHg)*
<1.5	Total Collapse	0–5
1.5–2.5	>50% Collapse	5–10
1.5–2.5	<50% Collapse	10–15
>2.5	<50% Collapse	15–20
>2.5	No Collapse	>20

IVC: inferior vena cava, RA: right atrial.

**Table 2:** Comparison of noninvasive techniques for hemodynamic monitoring.

Technique	Continuous	Operator dependent	Initial cost	Need for lead replacement	Supportive clinical literature for bedside hemodynamics	Correlation with TD
Ultrasound	N	+++	+++	N	+++	+++
TCDU	N	++	++	N	++	++
TEB	Y	+	++	Y	+++	++
BR	Y	+	++	Y	++	+++

Y: yes, N: No, +: fair, ++: moderate, +++: high.

TCDU: Transcutaneous Doppler ultrasound, TEB: Thoracic Electric Bioimpedance, BR: Bioreactance.
